# High-Linearity Wireless Passive Temperature Sensor Based on Metamaterial Structure with Rotation-Insensitive Distance-Based Warning Ability

**DOI:** 10.3390/nano13172482

**Published:** 2023-09-03

**Authors:** Chenying Wang, Luntao Chen, Bian Tian, Zhuangde Jiang

**Affiliations:** 1 School of Instrument Science and Technology, Xi’an Jiaotong University, Xi’an 710049, China; wangchenying@mail.xjtu.edu.cn; 2 State Key Laboratory for Manufacturing Systems Engineering, Xi’an Jiaotong University, Xi’an 710049, China; chenlt7676@stu.xjtu.edu.cn (L.C.); zdjiang@xjtu.edu.cn (Z.J.); 3 School of Mechanical Engineering, Xi’an Jiaotong University, Xi’an 710049, China

**Keywords:** temperature sensor, wireless passive, metamaterial structure, microwave scattering measurement

## Abstract

A wireless passive temperature sensor based on a metamaterial structure is proposed that is capable of measuring the temperature of moving parts. The sensor structure consists of an alumina ceramic substrate with a square metal double split-ring resonator fixed centrally on the ceramic substrate. Since the dielectric constant of the alumina ceramic substrate is temperature sensitive, the resonant frequency of the sensor is altered due to changes in temperature. A wireless antenna is used to detect the change in the resonant frequency of the sensor using a wireless antenna, thereby realizing temperature sensing operation of the sensor. The temperature sensitivity of the sensor is determined to be 205.22 kHz/°C with a strong linear response when tested over the temperature range of 25–135 °C, which is evident from the R2 being 0.995. Additionally, the frequency variation in this sensor is insensitive to the angle of rotation and can be used for temperature measurement of rotating parts. The sensor also has a distance warning functionality, which offers additional safety for the user by providing early warning signals when the heating equipment overheats after operating for extended durations.

## 1. Introduction

Temperature is an important physical parameter that is used for characterizing the state and properties of an object and plays an important role in daily life as well as in industrial production [1,2,3]. Commonly used temperature measurement methods utilize fiber-optics and thin film thermocouples that not only require a power supply and physical wired connections but also need to be able to operate in complex temperature measurement systems [4,5,6,7,8,9]. The application of wired active temperature sensors can be challenging in many applications, such as in rotating parts. Therefore, wireless passive measurements have been applied to temperature measurement by virtue of their ability to sense in real time without interference from long distance wiring and circuit interference.

There are different and quite mature techniques to measure the temperature in a contactless way. Hauser et al. proposed to use surface wave technology for passive, wireless magnetic impedance sensors and demonstrated its high efficiency [10]. As soon as MI strongly depends on the temperature for selected nanomaterials, it can be used as the temperature measurements [11]. There were also appropriate studies for magnetoelastic resonance cases and other attractive directions, such as wireless thermometers [12,13,14]. However, when using the above method for temperature measurement, the combined effects of material, temperature, and mechanical stress have to be considered. Meanwhile the temperature measurement range needs to be improved. Therefore, for moving parts, especially rotating parts, there is a need to investigate wireless passive temperature measurement methods that have a wide temperature range and are not affected by external forces.

The three basic wireless passive sensor technologies are coupled capacitive inductive near-field (LC) sensor technology, surface acoustic wave (SAW) sensing technology, and microwave backscattered signal readout (MST). The LC sensing technology has a closer readout distance and a lower quality factor [15,16]. Additionally, the results of the test can be affected by the presence of metal coils that generate eddy currents when the sensor is placed on a metal surface. The high substrate requirements and relatively low operating frequency of SAWs demand large-sized wireless interrogation antennas for wireless passive sensor devices [17,18]. Due to their high penetration ability, the transmission of microwaves is less disturbed by the environment, so MST has the advantages of high sensitivity and accuracy and can be used for temperature monitoring in complex environments [19,20].

Wireless passive temperature sensors using the MST principle have received increasing attention, which is evident from several demonstrations of such sensors in the literature. For instance, Sean Scott et al. [21] designed a double cantilever beam structure on the slot antenna as the sensor to realize the temperature measurement. However, their structure had a complex structure, which is challenging to fabricate. Cheng et al. [22] employed an integrated cylindrical resonator/antenna to create a large-scale wireless temperature sensor. Fei et al. [23] demonstrated a wireless temperature sensor with a relatively low sensitivity of 101.94 kHz/°C, which was made from an alumina-backed Au slot radiation patch. Subsequently, Tan et al. [24] utilized a double split-ring resonator structure based on a metamaterial unit cell, which utilized high-temperature co-fired ceramics (HTCC) technology. However, the sensor fabrication using their method is time-consuming and difficult to control.

In this paper, a high-linearity, wireless, rotation-insensitive, and passive temperature sensor is proposed with distance ranging function based on a metamaterial structure. The sensing principle is based on wireless MST sensors. The temperature sensitivity of the dielectric constant of the substrate causes the resonant frequency of the sensor to change. Passive and wireless temperature sensing is then performed by detecting the resonant frequency offsets. The temperature sensors are characterized by high linearity using a double split-ring resonator structure with a metamaterial cell. The fabrication of the sensor involves the sputtering of copper thin films on alumina ceramics using magnetron sputtering. This method is selected as it offers short a cycle time and good controllability of temperature sensor preparation. The temperature sensor is evaluated with a sensitivity of 205.22 kHz/°C and a linearity of R2 = 0.995 throughout a temperature spectrum that spans 25–135 °C. The proposed temperature sensor is utilized to test the temperature of static rotating parts, which lays the foundation for the next step of realizing the dynamic test. Additionally, the sensor is also capable of measuring distance, which provides the added benefit of long-distance heat alarms in case hazardous temperatures are encountered during operation.

## 2. Results and Discussion

### 2.1. Sensing Characteristics of Temperature Sensors

**Figure 1:**
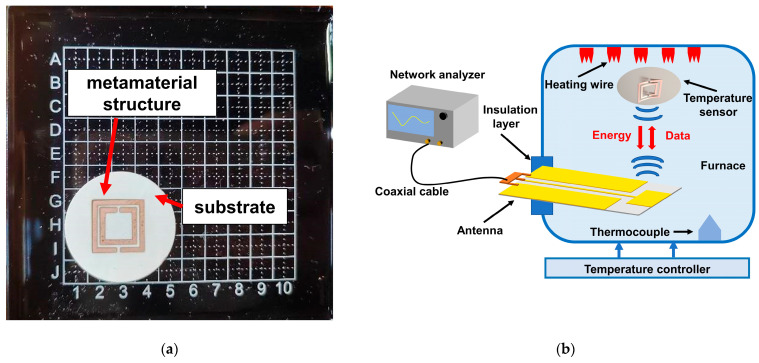

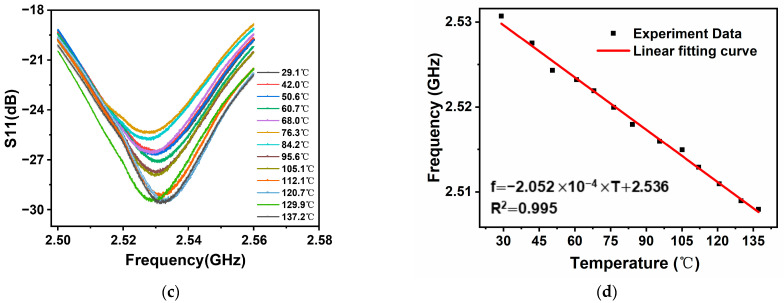
(**a**) Front view of the fabricated temperature sensor. (**b**) Temperature testing platform for wireless passive sensor. (**c**) Return loss measured by network analyzer at different temperatures. (**d**) Variation in the sensor’s resonant rate caused by temperature.

Figure 1a shows a physical diagram of the temperature sensor. The temperature sensor includes a substrate layer, which is a temperature-sensitive element whose dielectric constant is affected by temperature. The substrate layer is provided with a metamaterial structure, and the proposed metamaterial structure consists of a square-shaped double split-ring resonator structure, which forms a resonant structural unit. In this study, a metamaterial structure is used instead of the metal component in the surface plasmon resonant sensor to improve the sensitivity of the temperature sensor [25,26,27]. Due to the resonant characteristics of the double split-ring resonator structure, the sensor produces a sharp resonant frequency dip in its transmission spectrum. Furthermore, the double split-ring resonator structure has a high electric field density at the gap, which results in the sensor exhibiting excellent sensitivity and a highly linear temperature response. The metamaterial structure also acts as a reflective patch for wireless passive sensors, which generates an echo signal. The metamaterial structure is positioned at the center of the substrate in order to reduce the positioning error during processing, which ensures the processing accuracy of the sensor. The square double split-ring resonator structure (SDSRR) is made from copper, whereas the substrate layer material is composed of alumina ceramic, which is a dielectric material. Alumina ceramic’s dielectric constant fluctuates with temperature. The amount of translation of the peak of the return loss produced by the resonant structural unit determines the temperature sensor’s sensitivity. In order to facilitate a more pronounced temperature effect on the substrate layer due to external temperature, the substrate layer structure is chosen to be energy efficient in terms of heat transfer. As a result, a circular structure is selected, since it exhibits the least exothermic character.

To ensure the efficient use of wireless passive temperature sensors, a platform is fabricated for temperature experiments, according to the schematic illustration in Figure 1b. The interrogation antenna is a coplanar waveguide antenna. It uses a coaxial cable to connect to a network analyzer for wireless interrogation, to excite the temperature sensor, and to receive the temperature sensor’s return signal. Figure 1c depicts the temperature sensor’s return loss curves as recorded by the network analyzer at various temperatures. Finding the frequency at which the return loss curve reaches its maximum value yields the sensor’s resonant frequency. Figure 1d illustrates how changing the temperature alters the resonant frequency and yields a linear relationship. An equation of the following type can be used to fit the linear curve:

(1) $$f=-2.052\times 10^{-4}T+2.536,$$

where the correlation coefficient value (R2) is 0.995, *f* is the resonant frequency, and *T* is the temperature. The sensitivity of the sensor is determined to be 205.22 kHz/°C, while the temperature range being measured is 25 °C to 135 °C, and good linearity is observed.

### 2.2. Simulation Study of the Principle and Structure of the Temperature Sensor

**Figure 2:**
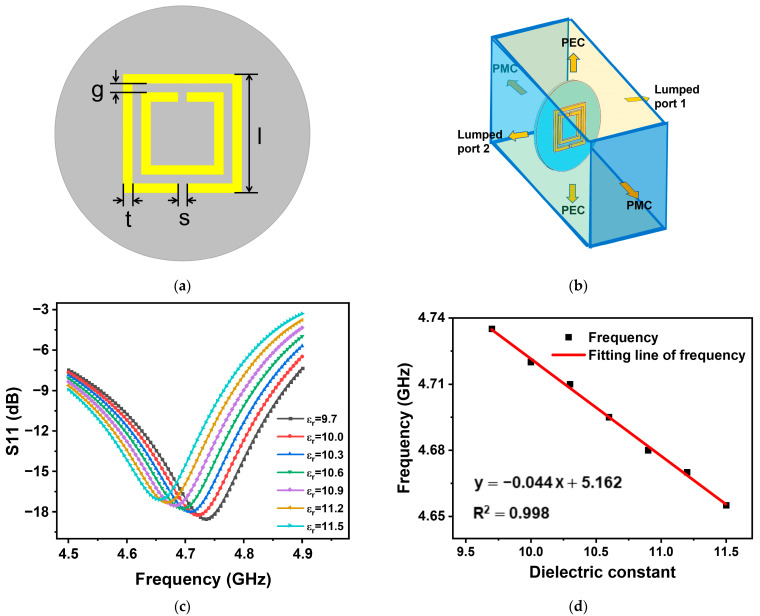
(**a**) An illustration of the sensor structure. (**b**) Sensor model schematic created using the COMSOL Multiphysics software. (**c**) The modeling shows the connection between sensor return loss and resonant frequency under different dielectric constants. (**d**) Simulated dielectric constant and resonant frequency relationship curve of the sensor.

The interrogation antenna is a crucial component of the wireless passive sensor for temperature measurement system. By design, the interrogation antenna’s swept frequency range ought to contain the sensor’s resonant frequency. In order to explore the frequency–temperature relationship of the wireless passive temperature sensor and the structural design of the sensor, the electromagnetic response of the sensor is modeled and analyzed using the simulation software COMSOL5.6. The depicted configuration of the temperature sensor can be observed in Figure 2a. The square double split-ring resonator structure has an outer ring length denoted by l, a ring width of t, and a ring opening width of s, with the spacing between the two rings being g. Figure 2b illustrates the vertical relationship between the sensor position and each boundary condition in the simulation.

We investigate the effects of altering the substrate layer’s dielectric constant on the sensor’s resonant frequency in the simulations. The information shows that the sensor frequency noticeably decreases as the substrate’s constant of dielectric rises. This correlation is illustrated in Figure 2c. This phenomenon is attributed to the decreased ordering of charges due to increased thermal motion at elevated temperatures. When the sensor is placed in an alternating electromagnetic field, polarized charges are generated in the substrate layer material. As the environmental temperature increases, the ions start to move more vigorously due to thermal energy. The information shows that the sensor frequency noticeably decreases as the substrate’s constant of dielectric rises. The strength of the electric field weakens, causing the capacitance of the sensor to increase. Higher temperatures cause more rapid changes in the electric field according to the following formula:

(2) $$f=\frac{1}{2\pi \sqrt{LC}},$$

Based on the equation given, where *f* represents the sensor frequency, *L* represents the sensor equivalent inductance, and *C* represents the sensor equivalent capacitance, we can conclude that, as the temperature increases, the resonant frequency of the sensor decreases. Figure 2d depicts the temperature measurement curve of the sensor, along with the following equation for the linear fitting curve:

(3) y=−0.044x+5.162,

which has a correlation coefficient of R2 = 0.998. It can be concluded that the simulation results do indeed predict a linear relationship between temperature and the resonant frequency of the sensor. The connection between temperature and the sensor’s resonant frequency is caused by fluctuations in the dielectric value of the dielectric material in the substrate layer. This relationship is linear, which means that when the temperature varies, so does the sensor’s resonant frequency. The variation in the sensor’s resonant frequency, which can signal variations in the ambient temperature, can be used to measure sensor temperature.

The SDSRR used in the sensor is based on a double split-ring resonator structure. In this structure, two open reverse notched metal square rings are combined together. Due to this design, the ring notch will accumulate charge to generate an induced capacitance. Furthermore, the gap between the two rings will also generate capacitance to accumulate charge. Due to the symmetry of the rings, the inter-ring electric dipole moments and the electric dipole moments generated at the notch will cancel each other [28]. As a result, the two notched square rings are combined to become an split-ring resonator. It has been shown that the resonant frequency of an split-ring resonator is related to the structural parameters of the split-ring resonator [29,30]. Since the structure as a transponder antenna, its resonant frequency affects the performance of wireless passive sensing. The structural parameters of the square double split-ring resonator are simulated using the single variable method. In the simulations, out of the four parameters (*l, t, s, g*), three parameters are fixed, and the relationship between the transducer frequency and the return loss under the variation of the fourth parameter is investigated.

**Figure 3:**
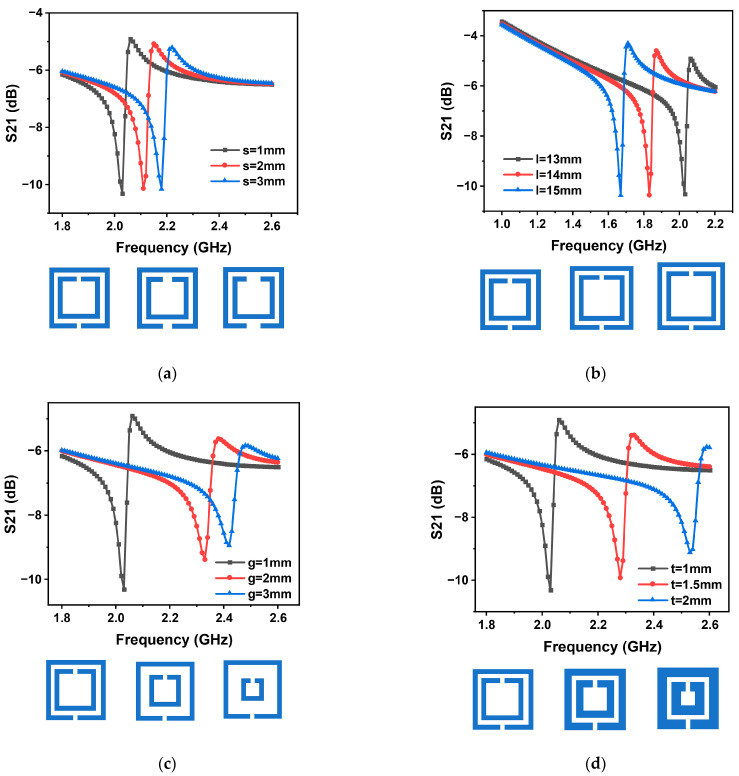
COMSOL simulation of (**a**) the width *s* of the SDSRR; (**b**) the length *l* of the SDSRR; (**c**) the width *g* of the SDSRR; (**d**) the width *t* of the SDSRR.

The simulation results show that the transducer’s resonant frequency drops when the value of s rises when the values of *l, t,* and g are held constant. This can be observed in Figure 3a. Under the conditions of fixed *t, g*, and *s*, as l is increased, the sensor’s resonant frequency falls, the return loss value rises, and the sharpness of the spike falls, as shown in Figure 3b. When *l, t*, and *s* are fixed, as *g* is increased, the resonant frequency of the sensor increases, the return loss value decreases, the sharpness of the spike decreases, and the sensor quality factor decreases, as shown in Figure 3c. Finally, for fixed *l, s*, and *g,* the data presented in Figure 3d illustrates that the resonant frequency of the sensor exhibits an upward trend as the value of *t* increases. Based on insights obtained from the simulations, the optimum performance is achieved by choosing a structure with *l* = 13 mm, *t* = 1 mm, *s* = 1 mm, and *g* = 1 mm. Under this dimensional configuration, the sensor exhibits the sharpest peak return loss, the smallest resonant frequency, and the highest sensitivity.

### 2.3. Investigating the Distance and Position between Sensor and Antenna in a Wireless Passive Temperature Testing System

**Figure 4:**
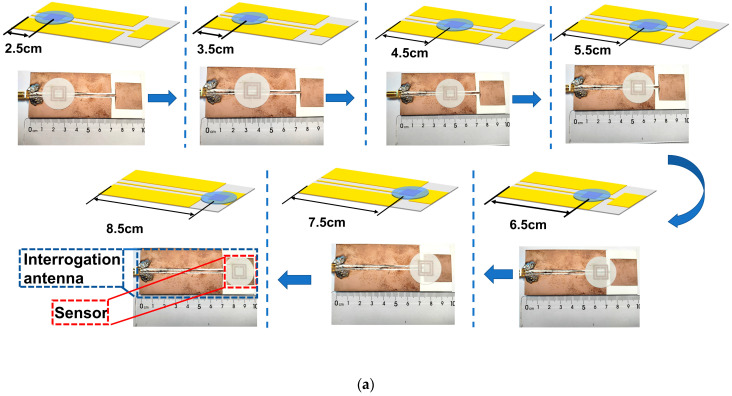

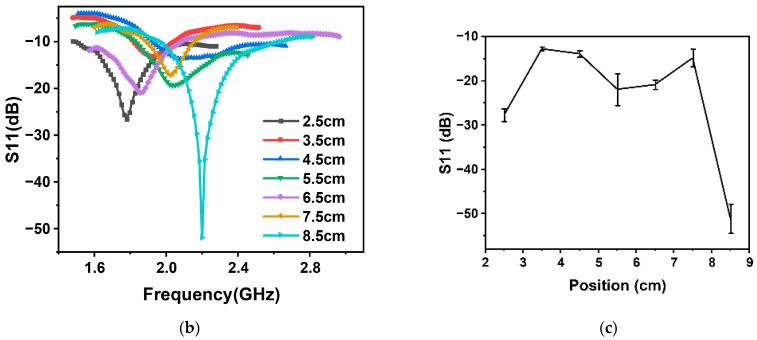
Experimental results of sensor and interrogation antenna positioning. (**a**) Diagram of sensor and interrogation antenna position test. (**b**) Experimental curve of the sensor in different positions. (**c**) Return loss of the sensor as a function of position.

The position and distance relationship between the sensor and the interrogating antenna is examined with the aim of enhancing the precision of the wireless passive temperature sensor’s temperature measurements. Figure 4a shows the configurations involved in testing the effect of position of the probing sensor and interrogating antenna. When the sensor is placed at a location within the plane of the interrogating antenna, there are multiple peaks in the return loss curve over the swept range. Because we are focusing on the offset of the peak with temperature when the sensor measures temperature, we chose to focus on the frequency point where the spikes are the sharpest and the return loss is the greatest. It is also found that multiple peaks of return loss in the swept range change when changing the position of the sensor in the interrogation antenna plane. That is, the corresponding maximum peaks of return loss are not the same when the sensor is at different positions in the interrogation antenna plane. Therefore, this experiment is to find the sharpest peaks of return loss when the sensor is located at different planar positions of the interrogating antenna, as shown in Figure 4b. Comparing the sharpest peaks of the return loss curves for each position, it can be seen that there is a difference in the return loss corresponding to these sharpest peaks. As shown in Figure 4c, by selecting the spikes with the maximum return loss among these spikes, it can be seen that the return loss at the point corresponding to the frequency at the position of 8.5 cm is the largest, and this position can make the peak of the curve more prominent, and at the same time, it means that the sensor has the best match with the interrogating antenna. Therefore, it can be concluded that the sensor should be placed at the far-right end of the interrogating antenna in the temperature test system. In this arrangement, the largest return loss value can be achieved. A number of experiments have been conducted, and the standard experimental error value is less than 3.5 dB [31].

**Figure 5:**
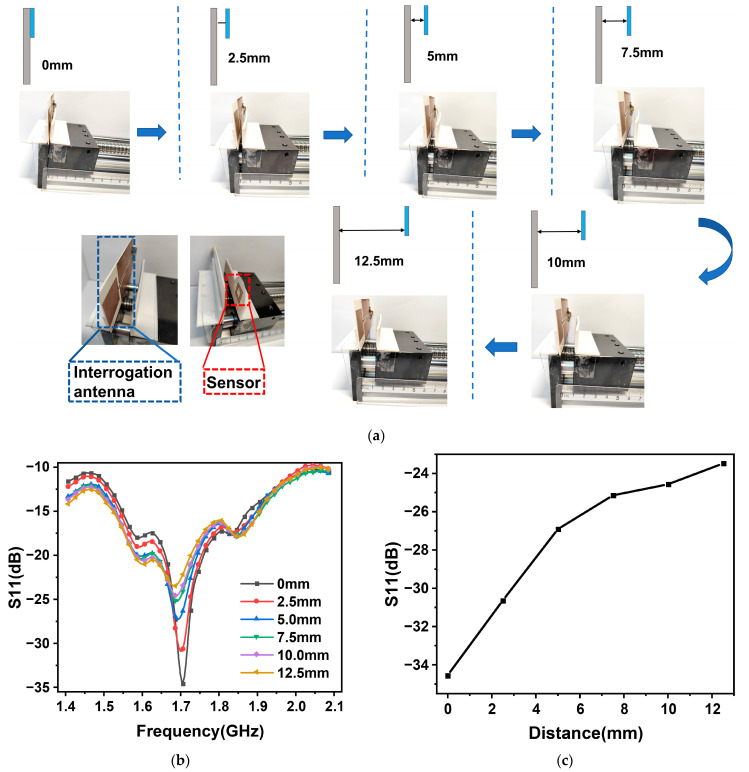
Experimental results of distance tests between sensor and interrogating antenna. (**a**) Diagram of the different test distance devices. (**b**) Return loss as function of frequency at different test distances. (**c**) Return loss of the sensor as a function of distance.

Once the position is optimized, the relationship between the distance from the sensor to the interrogation antenna and the return loss is explored at that location. Figure 5a shows the various configurations involved in the distance test experiments between the probe sensor and interrogation antenna. Results show that return loss decreases as the distance increases, as is evident from Figure 5b. Increasing the distance also results in a progressive weakening of the signal coupling, and deterioration of the transmission performance. Figure 5c demonstrates that when the sensor is situated close to the interrogating antenna, the wireless passive transmission has the maximum return loss. Based on these findings, the best spacing among the sensor and the interrogating antenna should be selected to reduce the transmission loss. This will allow the network analyzer to accurately show the resonant frequency information of the sensor. It is also possible to increase the interrogation distance by selecting interrogation antennas with superior performance, and good directionality that have a higher power transmission, thereby increasing the temperature measurement capability of the test system.

### 2.4. Wireless Passive-Based Temperature Sensor Temperature Measurement and Distance Warning Function

**Figure 6:**
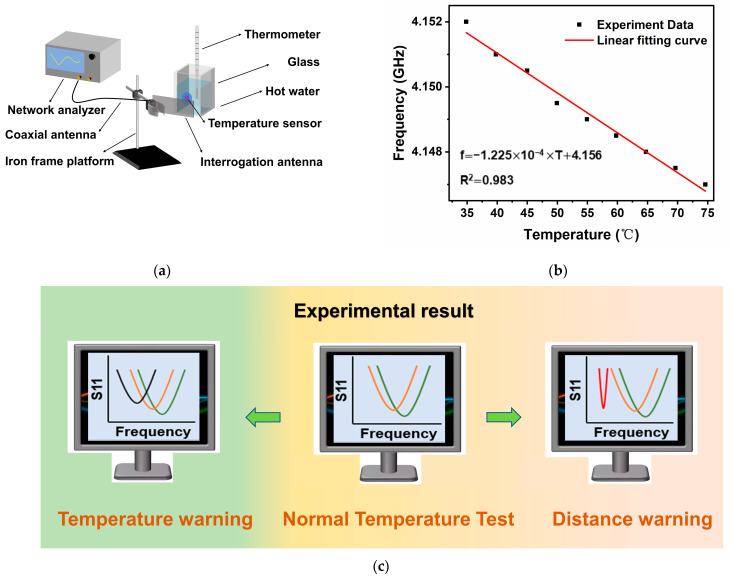

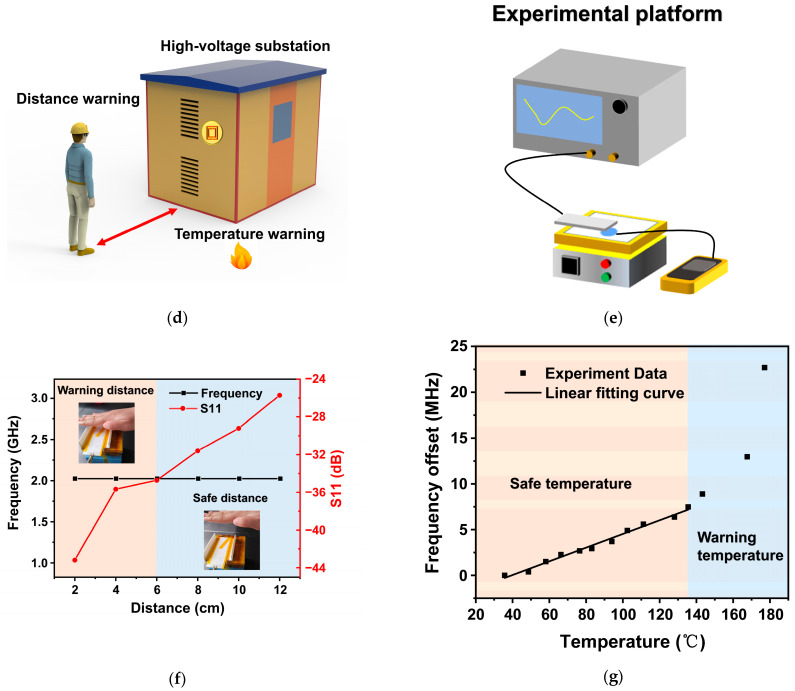
Practical applications of wireless and passive temperature sensors. (**a**) Sensor used to measure water temperature. (**b**) The resonant frequency of the sensor in relation to water temperature. (**c**) Diagrammatic representation of the distance-based temperature alarm function of the temperature sensor. (**d**) Diagrammatic representation of the distance-based temperature alarm function of a temperature sensor applied to a laboratory temperature testing system. (**e**) A concept sketch of a distance-based temperature warning system of a substation. (**f**) Diagram of the results of the distance alarm function of a temperature sensor applied to a laboratory temperature testing system. (**g**) Diagram of the result of a temperature alarm function of temperature sensor applied in a laboratory temperature testing system.

As a proof-of-concept, the fabricated temperature sensor was used to measure the water temperature. For this purpose, a wireless passive sensor water temperature test platform was constructed, as shown in Figure 6a. Results demonstrate a highly linear temperature sensing performance for the wireless passive sensor developed in this paper, where a correlation coefficient of R2 = 0.983 is obtained, as shown in Figure 6b. Figure 6c shows the frequency versus return loss curves of the sensor with temperature values displayed on the network analyzer in the temperature test experiment. This configuration allows for testing of the ambient temperature as well as the determination of the distance of a person close to the sensor. Based on this approach, wireless passive temperature sensors can be used for generating temperature warnings at a distance from the sensor that is mounted on to high voltage power equipment, as shown in the concept sketch in Figure 6e. With advancements in sensing technology, it is envisaged that a worker can not only obtain the temperature but also be made aware of hazardous temperatures using an early warning system. In the absence of safety personnel, such a system can alert workers when approaching dangerous equipment to avoid accidents and economic losses.

Figure 6d shows a laboratory simulation of the distance-based temperature sensor warning function. A subject approaches the sensor by placing their hand close to the sensor. The peak return loss curve experiences a greater effect if the hand is closer to sensor. The resonant frequency is maintained at 2.03 GHz, as depicted in Figure 6f. If the warning distance is configured to be 6 cm between the hand and the sensor, then the warning distance can be reached by judging that the return loss value of the frequency peak is greater than 34 dB and, at this time, the experimenter needs to pay attention to keep a safe distance from the heat source. Figure 6g shows that the change in temperature causes the resonant frequency shift of the temperature sensor to occur. In particular, a resonant frequency offset greater than 7.47 MHz can be used to alert the subject of possible contact with hazardous temperatures if the warning temperature is set to 135 °C.

### 2.5. Testing of Wireless Passive Temperature Sensors Acting on Rotating Blades

**Figure 7:**
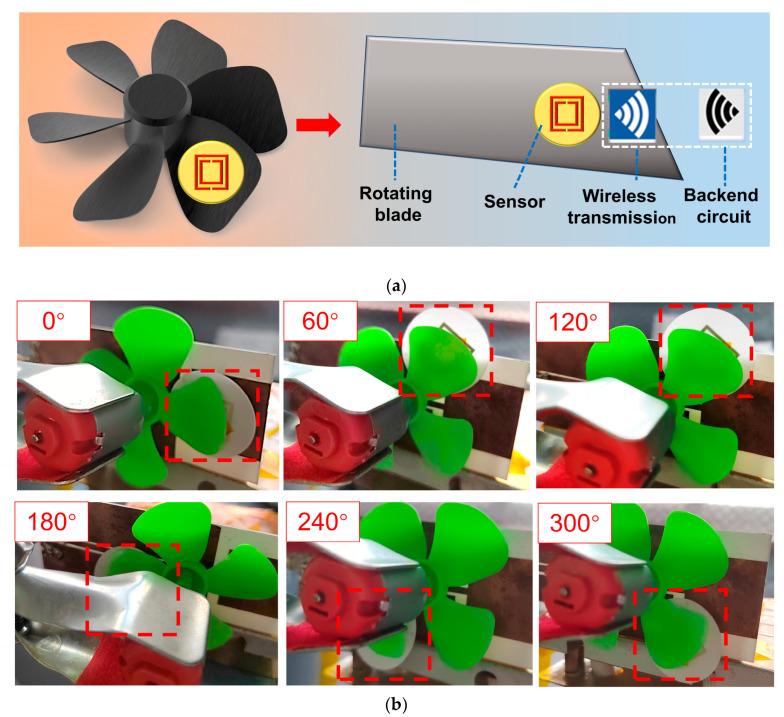

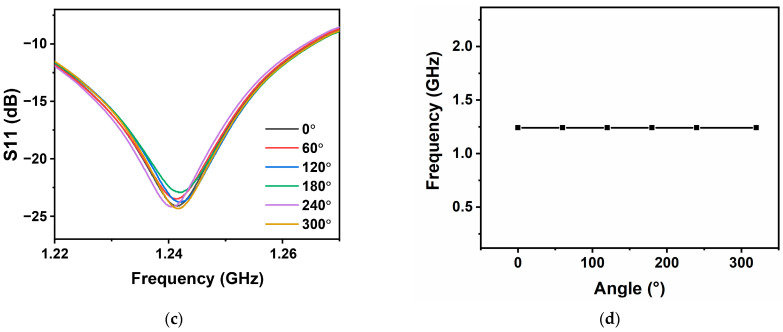
Practical application of the sensor. (**a**) Schematic of the wireless passive temperature sensor used for measuring blade temperature. (**b**) Experimental schematic of the wireless passive temperature sensor as applied to rotating blades (red dashed boxes indicate the sensor). (**c**) Return loss curves of the sensor at room temperature at various rotation angles on a rotating blade. (**d**) The resonant frequency dependence of the sensor rotation angle on the rotating blade at ambient temperature.

Conventional temperature sensors are not suitable for temperature measurement of rotating parts because it is challenging to attach leads to such parts. However, a wireless passive temperature sensor, like the one designed in this paper, can be used for temperature measurements of rotating blades. Figure 7a depicts the schematic of the wireless passive temperature sensor utilized to gauge leaf temperature. The sensor used in this experiment is attached to the blade and the blade temperature is fed to a computer as the wireless signal as shown in Figure 7b. Although the sensor experiments are carried out on a rotating blade, we only study the variation of one spike of a return loss curve. The return loss curves corresponding to blade rotation at different angles are recorded, as shown in Figure 7c. Then, the peak value of each curve corresponding to the resonant frequency value is extracted, as shown in Figure 7d. It is observed that the resonant frequency value at different angles is 1.241 GHz. In other words, the sensor’s resonant frequency is not affected by the rotation angle, making it suitable for detecting temperatures of moving parts. Wireless passive temperature sensors require an antenna for signal transmission. When measuring the temperature of rotating parts, it is important to consider the influence of environmental factors, such as wind speed, on the temperature measurement system. Follow-up research is required to study the return loss versus frequency of the sensor under conditions of blade rotation to investigate the effect of rotational speed, wind speed, and other factors on the sensor’s temperature measurement under blade rotation. Such a sensor has immense potential in aerospace applications.

## 3. Conclusions

In conclusion, a wireless passive temperature sensor that uses a metamaterial structure is created, demonstrating rotation-insensitive temperature detection as well as a distance-based warning capability. The sensor utilizes the MST wireless transmission principle, which provides an added advantage of being unaffected by environmental interference. The sensor adopts a square double split-ring resonator structure, which confers the sensor with high linearity. Over the temperature span between 25–135 °C, a sensitivity of 205.22 kHz/°C is found with a linearity of R2 = 0.995. The linear link between the sensor’s resonant frequency and temperature is confirmed by simulation findings that are in agreement with the results of trials. The simulations show that the dimensional parameters of the SDSRR affect the sensor’s resonant frequency shift, which affects the sensor’s sensitivity. Optimized structure parameters are obtained from simulations, where the outer ring edge length, the ring width, the ring opening width, and spacing between the two rings of the structure are chosen to be 13 mm, 1 mm, 1 mm, and 1 mm, respectively. The sensor should be placed at the rightmost end of the interrogating antenna during the temperature test experiments and kept at a close distance from the interrogating antenna, as it has been discovered that these factors have an impact on the accuracy of the temperature test results. In addition, several application scenarios for wireless passive temperature sensors are demonstrated. Wireless passive temperature sensors can be used in high-voltage hazardous power equipment to report the temperature when a subject is at a safe distance from the equipment, thus, improving the safety of handling equipment in hazardous situations. Wireless passive sensors are insensitive to the angle of rotation under rotating conditions and are expected to be used for temperature measurement of rotating parts, which is a requirement in the aerospace industry.

## 4. Experimental Component

An Agilent E5080B network analyzer is used for temperature sensing experiments. The interrogation antenna is a coplanar waveguide antenna, which is prepared by a magnetron sputtering process by sputtering metallic copper material on an alumina ceramic substrate. The SMA port on the interrogation antenna is soldered, and the interrogation antenna is coaxially attached to the network analyzer via the SMA port. The TR6602 thermocouple dual-channel K-type high-precision digital contact thermometer calibrates the temperature by wiring the sensor to directly detect the sensor’s temperature as the temperature coordinate value. For the water temperature tests, the antenna is fixed through the iron frame table, whereas the sensor is fixed on the outer side of the transparent glass cup. The electronic thermometer RE-W5007, which is submerged in water to obtain a direct reading of the water’s temperature as the temperature coordinate value, performs temperature calibration.

The sensor simulation is performed by applying special boundaries to the S-waveguide where the sensor is located. The ideal magnetic conductor (PMC) boundary is set perpendicular to the base plane of the sensor, while the ideal electric conductor (PEC) boundary and the collector port boundary correspond to the other two axes of the coordinate system, respectively. The three boundaries are set perpendicular to each other. Both platinum and alumina ceramics are high temperature resistant materials, and the simulation is set up with the substrate material being alumina ceramics and the metal ring material being platinum. The simulation is set up so that the dielectric constant of alumina ceramics rises linearly from 9.7 to 11.5 when the ambient temperature is made to range from 28 °C to 1100 °C.

For the location and distance testing, the ambient temperature is kept constant, and only the effect of moving the location as well as the distance among the sensor and the interrogation antenna on the return loss curve is explored. The sensor and interrogation antenna positions were tested experimentally by moving the sensor from the leftmost to the rightmost side of the interrogation antenna. The leftmost position corresponds to the reference position, and the data is marked at 2.5 cm. The interrogation antenna is fixed, whereas the sensor is moved in steps of 1 cm to the right end. The rightmost end of the data is recorded at 8.5 cm, as illustrated in Figure 4a. For the sensor and interrogation antenna distance experiments, the interrogation antenna is fixed, the sensor is mounted on the slide table, and the sensor position is directly to the rightmost end of the interrogation antenna. When the slide table is moved, the sensor and the interrogation antenna positions increase. Each time, the table is moved by 2.5 mm, from the tight fit to 12.5 mm, as shown in Figure 5a.

In the sensor rotation experiment, the interrogating antenna is kept stationary, the sensor is fixed on one of the rotating blades, and the initial position of the sensor is kept directly opposite to the interrogating antenna and at the rightmost end of the interrogating antenna, which is noted to have a rotation angle of 0°. The blades are rotated, and a graph is recorded of return loss versus frequency in the network analyzer at rotation angles of 0°, 60°, 120°, 180°, 240°, and 320°.

## Data Availability

Data sharing does not apply to this article.
